# Association between type 2 diabetes and depressive symptoms after a 1-year follow-up in an older adult Mediterranean population

**DOI:** 10.1007/s40618-023-02278-y

**Published:** 2024-01-13

**Authors:** I. Baenas, L. Camacho-Barcia, R. Granero, C. Razquin, D. Corella, C. Gómez-Martínez, O. Castañer-Niño, J. A. Martínez, Á. M. Alonso-Gómez, J. Wärnberg, J. Vioque, D. Romaguera, J. López-Miranda, R. Estruch, F. J. Tinahones, J. Lapetra, J. L. Serra-Majem, N. Cano-Ibáñez, J. A. Tur, V. Martín-Sánchez, X. Pintó, J. J. Gaforio, P. Matía-Martín, J. Vidal, C. Vázquez, L. Daimiel, E. Ros, S. Jiménez-Murcia, S. Dalsgaard, A. Garcia-Arellano, N. Babio, J. V. Sorli, C. Lassale, M. García-de-la-Hera, E. Gómez-García, M. A. Zulet, J. Konieczna, S. Martín-Peláez, L. Tojal-Sierra, F. J. Basterra-Gortari, S. de las Heras-Delgado, O. Portoles, M. Á. Muñoz-Pérez, A. P. Arenas-Larriva, L. Compañ-Gabucio, S. Eguaras, S. Shyam, M. Fitó, R. M. Baños, J. Salas-Salvadó, F. Fernández-Aranda

**Affiliations:** 1 Eating Disorders Unit, Clinical Psychology Department, University Hospital of Bellvitge, Feixa Llarga s/n, Hospitalet de Llobregat, 08907 Barcelona, Spain; 2 Centro de Investigación Biomédica en Red, Fisiopatología de la Obesidad y la Nutrición (CIBERobn), Instituto de Salud Carlos III, 28029 Madrid, Spain; 3 Psychoneurobiology of Eating and Addictive Behaviors Group, Neurosciences Programme, Bellvitge Biomedical Research Institute—IDIBELL, 08908 Barcelona, Spain; 4 Doctoral Program in Medicine and Translational Research, University of Barcelona, 08007 Barcelona, Spain; 5 Department de Psicobiologia I Metodologia de les Ciències de la Salut, Universitat Autònoma de Barcelona, 08193 Barcelona, Spain; 6 Department of Preventive Medicine and Public Health, University of Navarra, IDISNA, 31008 Pamplona, Spain; 7 Department of Preventive Medicine, University of Valencia, 46010 Valencia, Spain; 8 Human Nutrition Unit ANUT-DSM, Biochemistry and Biotechnology Department, Faculty of Medicine and Health Sciences, Universitat Rovira i Virgili, C/Sant Llorenç 21, 43201 Reus, Spain; 9 Institut d’Investigació Sanitària Pere Virgili (IISPV), 43007 Reus, Spain; 10 Unit of Cardiovascular Risk and Nutrition, Institut Hospital del Mar de Investigaciones Médicas Municipal d`Investigació Médica (IMIM), 08003 Barcelona, Spain; 11 CIBER de Epidemiología y Salud Pública (CIBEResp), Instituto de Salud Carlos III, 28029 Madrid, Spain; 12 Department of Nutrition, Food Sciences, and Physiology, Center for Nutrition Research, University of Navarra, 31008 Pamplona, Spain; 13 Precision Nutrition and Cardiometabolic Health Program, IMDEA Food, CEI UAM + CSIC, 28049 Madrid, Spain; 14 Cardiovascular, Respiratory and Metabolic Area, Bioaraba Health Research Institute, Osakidetza Basque Health Service, Araba University Hospital, University of the Basque Country UPV/EHU, 01009 Vitoria-Gasteiz, Spain; 15 Department of Nursing, University of Málaga, Institute of Biomedical Research in Malaga (IBIMA), 29590 Málaga, Spain; 16 Instituto de Investigación Sanitaria y Biomédica de Alicante, Universidad Miguel Hernández (ISABIAL-UMH), 03010 Alicante, Spain; 17 Health Research Institute of the Balearic Islands (IdISBa), University Hospital Son Espases, 07120 Palma, Spain; 18 Department of Internal Medicine, Maimonides Biomedical Research Institute of Cordoba (IMIBIC), Reina Sofia University Hospital, University of Cordoba, 14004 Cordoba, Spain; 19 Department of Internal Medicine, Institut d’Investigacions Biomèdiques August Pi Sunyer (IDIBAPS), Hospital Clinic, University of Barcelona, 08036 Barcelona, Spain; 20 Department of Endocrinology, Instituto de Investigación Biomédica de Málaga (IBIMA), Virgen de la Victoria Hospital, University of Málaga, 29590 Málaga, Spain; 21 Department of Family Medicine, Research Unit, Distrito Sanitario Atención Primaria Sevilla, 41013 Seville, Spain; 22 Research Institute of Biomedical and Health Sciences (IUIBS), University of Las Palmas de Gran Canaria and Centro Hospitalario Universitario Insular Materno Infantil (CHUIMI), Canarian Health Service, 35016 Las Palmas de Gran Canaria, Spain; 23 Department of Preventive Medicine and Public Health, University of Granada, 18071 Granada, Spain; 24 Instituto de Investigación Biosanitaria de Granada (IBS.GRANADA), 18012 Granada, Spain; 25 Research Group on Community Nutrition and Oxidative Stress, University of Balearic Islands, 07122 Palma, Spain; 26 Institute of Biomedicine (IBIOMED), University of León, 24071 León, Spain; 27 Lipids and Vascular Risk Unit, Internal Medicine, Hospital Universitario de Bellvitge-IDIBELL, Universidad de Barcelona, 08908 Barcelona, Spain; 28 Departamento de Ciencias de la Salud, Instituto Universitario de Investigación en Olivar y Aceites de Oliva, Universidad de Jaén, 23071 Jaén, Spain; 29 Department of Endocrinology and Nutrition, Instituto de Investigación Sanitaria Hospital Clínico San Carlos (IdISSC), 28040 Madrid, Spain; 30 CIBER Diabetes y Enfermedades Metabólicas (CIBERDEM), Instituto de Salud Carlos III (ISCIII), 28029 Madrid, Spain; 31 Department of Endocrinology, Institut d` Investigacions Biomédiques August Pi Sunyer (IDIBAPS), Hospital Clinic, University of Barcelona, 08036 Barcelona, Spain; 32 Department of Endocrinology and Nutrition, Hospital Fundación Jimenez Díaz, Instituto de Investigaciones Biomédicas IISFJD. University Autonoma, 28024 Madrid, Spain; 33 Departamento de Ciencias Farmacéuticas y de la Salud, Facultad de Farmacia, Universidad San Pablo-CEU, CEU Universities, 28668 Madrid, Spain; 34 Department of Endocrinology and Nutrition, Institut d’Investigacions Biomèdiques August Pi Sunyer (IDIBAPS), Lipid Clinic, Hospital Clínic, 08036 Barcelona, Spain; 35 Department of Clinical Sciences, School of Medicine and Health Sciences, University of Barcelona, 08907 Barcelona, Spain; 36 NCRR—National Centre for Register-Based Research, Aarhus University, 8210 Aarhus, Denmark; 37 iPSYCH—The Lundbeck Foundation Initiative for Integrative Psychiatric Research, 8210 Aarhus, Denmark; 38 CIRRAU—Centre for Integrated Register-Based Research, Aarhus University, 8210 Aarhus, Denmark; 39 Barcelona Institute for Global Health (ISGlobal), 08036 Barcelona, Spain; 40 Department of Endocrinology and Nutrition, Hospital Universitario de Navarra, IdiSNA, Universidad Pública de Navarra, 31008 Pamplona, Spain; 41 Unitat de Suport a la Recerca en Atenció Primaria de Barcelona. IDIAP Jordi Gol. Primary Care Division, Institut Català de La Salut, 08007 Barcelona, Spain; 42 Department of Personality, Evaluation and Psychological Treatment of the University of Valencia, 46010 Valencia, Spain

**Keywords:** Type 2 diabetes, Depressive symptoms, Metabolic syndrome, HbA1c, Severity

## Abstract

**Objectives:**

To examine the cross-sectional association between baseline depressive symptoms and the presence of type 2 diabetes (T2D), and its association with glycated hemoglobin (HbA1c) and other metabolic variables, and the prospective association of depressive symptoms and HbA1c after 1 year of follow-up.

**Methods:**

*n* = 6224 Mediterranean older adults with overweight/obesity and metabolic syndrome (48% females, mean age 64.9 ± 4.9 years) were evaluated in the framework of the PREDIMED-Plus study cohort. Depressive symptoms were assessed using the Beck Depression Inventory-II and HbA1c was used to measure metabolic control.

**Results:**

The presence of T2D increased the likelihood of higher levels of depressive symptoms (*χ*^2^ = 15.84, *p* = 0.001). Polynomial contrast revealed a positive linear relationship (*χ*^2^ = 13.49, *p* = 0.001), the higher the depressive symptoms levels, the higher the prevalence of T2D. Longitudinal analyses showed that the higher baseline depressive symptoms levels, the higher the likelihood of being within the HbA1c ≥ 7% at 1-year level (Wald-*χ*^2^ = 24.06, *df* = 3, *p* < .001, for the full adjusted model). Additionally, depressive levels at baseline and duration of T2D predicted higher HbA1c and body mass index, and lower physical activity and adherence to Mediterranean Diet at 1 year of follow-up.

**Conclusions:**

This study supports an association between T2D and the severity of depressive symptoms, suggesting a worse metabolic control from mild severity levels in the short–medium term, influenced by lifestyle habits related to diabetes care. Screening for depressive symptoms and a multidisciplinary integrative therapeutic approach should be ensured in patients with T2D.

**Supplementary Information:**

The online version contains supplementary material available at 10.1007/s40618-023-02278-y.

## Introduction

Diabetes is a highly prevalent medical condition and a major health burden, affecting approximately 422 million people worldwide [1]. It is considered a leading cause of mortality and disability and is directly responsible for approximately 1.5 million deaths each year [1]. Type 2 diabetes (T2D) predominates in most cases, with a typical onset in adulthood. This metabolic disease has a complex etiology, involving multiple risk factors from genetics to environmental features, such as a sedentary lifestyle, malnutrition, and the presence of other metabolic disorders. Individuals with metabolic syndrome (MetS) and obesity are at an increased risk of developing T2D, which are often comorbid conditions [2]. Remarkably, these disorders are associated with multisystemic consequences in the short and middle-long term [1]. In addition to somatic complications, it is worth mentioning their impact on emotional well-being [3].

The presence of a chronic disease such as T2D implies a multifaceted psychological adjustment encompassing emotional, cognitive, and behavioral spheres. This process has a dynamic nature and is influenced by multiple disease-related and individual factors which, in turn, could modulate the individual’s capacity to adaptively adjust [4]. In this context, coping with a chronic disease and its consequences, the burden of self-care to maintain proper metabolic control and prevent the development of complications, as well as physical disability and social difficulties could contribute to a disorder-related emotional distress (i.e., diabetes-related distress) [5]. The presence of T2D also increases the vulnerability to the development of mood disturbances, from subthreshold symptoms to mood disorders [6, 7]. Accordingly, the meta-analysis by Harding et al. [8] described a prevalence of depressive symptoms ranging from 13.9 to 66.4% in patients with T2D. Furthermore, the prevalence of major depressive disorder (MDD) oscillates between 0.9 and 51.8% [9]. Depressive symptomatology in individuals with T2D has been associated with younger age and higher glycated hemoglobin (HbA1c) levels, leading to a higher risk of diabetic complications [8]. Alternatively, a higher vulnerability for a comorbid MDD has been associated with older age and higher weight, as well as with other comorbid chronic diseases [10]. In this context, MetS may contribute to an increased risk of mood disturbances in patients with T2D [7].

Due to the usual coexistence of metabolic and mood disturbances, some authors have suggested the existence of a so-called “metabolic-mood syndrome” [11], and common underlying neurobiological mechanisms and environmental factors between T2D, MetS, and mood disturbances have been suggested [12]. That said, the chronic proinflammatory state associated with MetS and obesity favors processes, such as hyperinsulinemia, lower tolerance to glucose, and dysregulation of the hypothalamus–pituitary–adrenal (HPA) axis, which may precede the development of T2D [13]. Likewise, this proinflammatory scenario and oxidative stress have been linked to the pathogenesis of depression [6], with a consequent increased cardiovascular risk [14]. On the other hand, unhealthy dietary habits, a sedentary lifestyle, and lower self-care are common environmental characteristics found in both individuals with mood disturbances and those with T2D [15].

Therefore, individuals with T2D represent a group of particular interest when analyzing the bidirectional relationship with mood-related symptomatology and the consequences derived from their comorbidity [13]. From diabetes-related distress to depression, the presence of mood disturbances may influence the management of the disorder in terms of metabolic control (e.g., HbA1c), adherence to diet and physical activity, or monitoring of diabetes-related complications, leading to increased morbidity and mortality and poorer quality of life [3]. This fact acquires a special significance in the middle adulthood and older adults, who represent a particularly vulnerable population for the confluence of both metabolic and mood anomalies, contributing to a higher cardiovascular risk and associated complications, such as cognitive impairment [14, 16]. Precisely, previous research developed in the context of the PREDIMED-Plus study examining older adults with T2D and MetS found that, in addition to poorer metabolic control and higher body mass index (BMI), depressive symptoms were associated with poorer neurocognitive status [17]. Some studies have analyzed strategies to ameliorate the comorbidity between metabolic and mood disturbances. In this regard, the beneficial effects of a Mediterranean Diet (MedDiet) intervention have been highlighted [15, 18–21].

Considering this, a few studies have investigated the link between depressive symptoms and T2D in an older adult population with MetS and a MedDiet intervention. The first objective of this research was to examine cross-sectionally the association between the severity levels of depressive symptoms and the presence of T2D. Second, among individuals with T2D, we explored whether depressive symptoms were related to metabolic control (i.e., HbA1c) and other MetS-related variables at baseline. Third, the link between baseline levels of depressive symptoms and HbA1c at 1-year follow-up was also assessed. Using path analysis, we evaluated the predictive capacity of baseline depressive symptoms on HbA1c at 1-year follow-up. This model also aimed to explore the potential mediating role of different features, such as T2D duration (at baseline), physical activity, adherence to MedDiet, and BMI (at 1-year follow-up). We expected to find higher levels of depressive symptoms in individuals with T2D. We also hypothesized that more severe depressive symptoms would be linked to a worse metabolic control both at baseline and at 1-year follow-up. Furthermore, we presumed that higher levels of depressive symptoms at baseline would predict a worse metabolic control at 1-year follow-up.

## Materials and methods

### Study design and participants

The present study was conducted in the context of the PREDIMED‐Plus study, a 6-year, controlled, randomized parallel-group intervention study. The main aim of this multicentric project is to assess the effects of an intensive lifestyle intervention based on an energy reduced MedDiet, physical activity promotion, and behavioral support on primary prevention of cardiovascular disease events. Participants were enrolled between 2013 and 2016 by 23 Spanish centers from different universities, hospitals, and research institutes working in collaboration with 208 Primary Care Health Facilities belonging to the Spanish National Health System. The total cohort included older adults (*n* = 6874), with men aged between 55 and 75 years, and women between 60 and 75 years. All the participants had overweight or obesity and met at least three components of MetS at the moment of enrollment [22]. They were randomly assigned to either an intensive weight loss intervention group, based on an energy reduced MedDiet with physical activity promotion and behavioral support, or to a control group, advised to follow an ad libitum MedDiet without any other indication. More details of the study, inclusion and exclusion criteria, and methods have been previously specified [23], and the protocol is available at http://predimedplus.com/. The PREDIMED‐Plus Study was registered at the International Standard Randomized Controlled Trial in 2014 (ISRCT; http://www.isrctn.com/ISRCTN89898870). According to the ethical standards of the Declaration of Helsinki by the Research Ethics Committees, the study protocol and procedures were approved from all the participating institutions. All participants provided written informed consent.

The sub-sample analyzed in this study comprised 6224 individuals at baseline (*n* = 1913 with prevalent T2D), as 650 participants were excluded from the total sample due to missing data on the variables assessed in this work. At 1-year follow-up, the sample was composed of 5559 individuals, after excluding 665 subjects due to incomplete data or dropout. Among them, *n* = 1694 had T2D. Supplementary Fig. 1 (S1) shows the sampling flowchart.

### Outcomes and assessments

#### Depressive symptoms’ evaluation

The Beck Depression Inventory–II (BDI-II) [24]; Spanish validation [25]: it is a 21-item self-report measure for assessing the severity of depressive symptoms in adults and adolescents (ages from 13 to 80 years). Scores for each item ranged from 0 to 3 and a total score is obtained from the sum of all responses. Higher total scores indicate more severe depressive symptomatology. The standardized cut-offs are as follows: 0–13 indicates minimal levels, 14–19 mild levels, 20–28 moderate levels, and 29–63 severe levels. The Cronbach’s alpha in our sample was α = 0.884.

#### Assessment of glycemic control

Fasting peripheral blood samples were obtained to determine HbA1c, that was used as a measure of metabolic control in T2D, establishing a cut-off point (7%) according to the American Diabetes Association (ADA) [26].

#### Anthropometric, blood pressure, and other biochemical measures

Weight, height, and waist circumference were measured in duplicate following a pre-established protocol. BMI (Kg/m^2^) was calculated by dividing the weight (Kg) by the square of height (m^2^). Waist circumference was determined midway between the lowest rib and the iliac crest. Blood pressure (mm/Hg) was measured with a validated semi‐automatic oscillometer (Omron HEM‐705CP). Fasting peripheral blood samples were obtained to determine serum glucose (mg/dl), insulin (mcU/dl), and lipid profile (mg/dl) [i.e., total cholesterol, high‐density lipoprotein cholesterol (HDLc), and triglycerides] using standard enzymatic methods. Low‐density lipoprotein cholesterol (LDLc) was calculated by the Friedwald formula whenever triglycerides were less than 300 mg/dl. Laboratory personnel performing all assays was blinded to group allocation.

#### Leisure-time physical activity and adherence to MedDiet

Adherence to the MedDiet pattern was assessed using the energy-restricted Mediterranean Diet Adherence Screener (er-MEDAS), a validated 17-item questionnaire [27]. This screener is a modified version from the previously validated Mediterranean Diet Adherence Screener (MEDAS), developed and applied in the framework of the randomized clinical trial PREDIMED Study. The er-MEDAS encompasses the 14 questions related to food consumption and behaviors from MEDAS, and 3 additional items aiming at capturing the dimension of energy restriction. Each of the items was scored as either 1 or 0, with a range between 0 and 17 points, 17 being the maximum adherence.

The Minnesota-REGICOR leisure-time physical activity questionnaire was used to estimate the physical activity total energy expenditure [28].

#### Other covariates

At baseline, trained staff collected participants’ information through face-to-face interviews regarding sociodemographic characteristics (i.e., sex, age, level of education, marital status, and employment status), T2D illness duration, and use of diabetic medication.

### Statistical analysis

Data analysis was performed with the Stata17 program for Windows [29]. The March 2019 PREDIMED-Plus database was used. Cross-sectional associations of the severity groups regarding depressive symptoms and presence of T2D at baseline, with the sociodemographic and clinical profiles, were performed with Chi-squared tests for categorical measures and with analysis of variance (ANOVA) for quantitative measures. The longitudinal association between the severity of depressive symptoms at baseline and HbA1c levels at 1-year follow-up was analyzed with a multivariate regression model, adjusting for the covariates HbA1c baseline levels, the data collection center, and the intervention group.

Path analysis was used to assess the predictive capacity of the duration of T2D and the levels of depressive symptomatology at baseline on the results registered at 1 year of follow-up in HbA1c, energy expenditure in leisure-time physical activity, adherence to MedDiet, and BMI. In this work, path analysis was implemented through Structural Equation Modeling (SEM), adjusted by HbA1c at baseline, the center, and the intervention group. The following procedure was used: (a) all parameters were free estimated (no initial values were assumed); (b) only statistical parameters were retained in the final model to achieve the most parsimonious model and facilitate interpretation; and (c) the maximum-likelihood estimation method was used. Goodness-of-fit was evaluated using the typical fit indexes/criteria: Root-Mean-Square Error of approximation RMSEA < 0.08, Bentler’s Comparative Fit Index CFI > 0.90, Tucker–Lewis Index TLI > 0.90, and Standardized Root-Mean-Square Residual SRMR < 0.10. The global predictive capacity of the model was measured by the coefficient of determination (CD).

In this study, Finner's method was applied to control the increase in the Type I error due to the performance of multiple null-hypothesis tests [30].

## Results

### Characteristics of the participants

Table 1 shows the descriptive for the sample at baseline (*n* = 6224). The mean age of the sample was 64.9 years (SD = 4.9 years) and 52% of the participants were men (*n* = 3239). Participants were predominantly married (76.3%), with education up to the primary level (47.7%) and retired from active service (58%). Regarding clinical features, 49.3% of the sample had obesity type I, followed by overweight (26.6%) and obesity type II (23.3%). Most of the participants had HbA1c < 7% (87.4%) and 30.7% suffered from T2D. While 79.8% of the cases reported “minimal depressive symptoms”, 11.1% of the participants reported “mild depressive symptoms”, 6.9% “moderate depressive symptoms”, and 2.2% “severe depressive symptoms”.

**Table 1:** Baseline characteristics of the sample

	*n* = 6224	
	*n*	%
Sex		
Men	3239	52.0
Women	2985	48.0
Marital status		
Single	344	5.5
Married	4751	76.3
Divorced-separated	500	8.0
Widowed	629	10.1
Education level		
Higher education	1387	22.3
Secondary	1815	29.2
Primary	2969	47.7
Less than primary	53	0.8
Employment status		
Employed	1344	21.6
Work at home	903	14.5
Retired	3613	58.0
Unemployed (with incomes)	241	3.9
Unemployed (no incomes)	123	2.0
Weight status		
Overweight	1657	26.6
Obesity I (BMI 30–34.9)	3066	49.3
Obesity II or more (BMI ≥ 35)	1501	24.2
Depressive symptoms		
Minimal (score 0–13)	4967	79.8
Mild (score 14–19)	688	11.1
Moderate (score 20–28)	431	6.9
Severe (score ≥ 29)	138	2.2
HbA1c		
Low-normal (< 7.0%)	5438	87.4
High (≥ 7.0%)	786	12.6
T2D		
Absent	4311	69.3
Present	1913	30.7
Age (years)	Mean	SD
	64.9	4.9

Depressive symptoms, BDI-II scores: minimal (0–13), mild (14–19), moderate (20–28), and severe (29 or high). *SD* standard deviation, *HbA1c* glycosylated hemoglobin, *T2D* type 2 diabetes

### Cross-sectional analyses

**Fig. 1:**
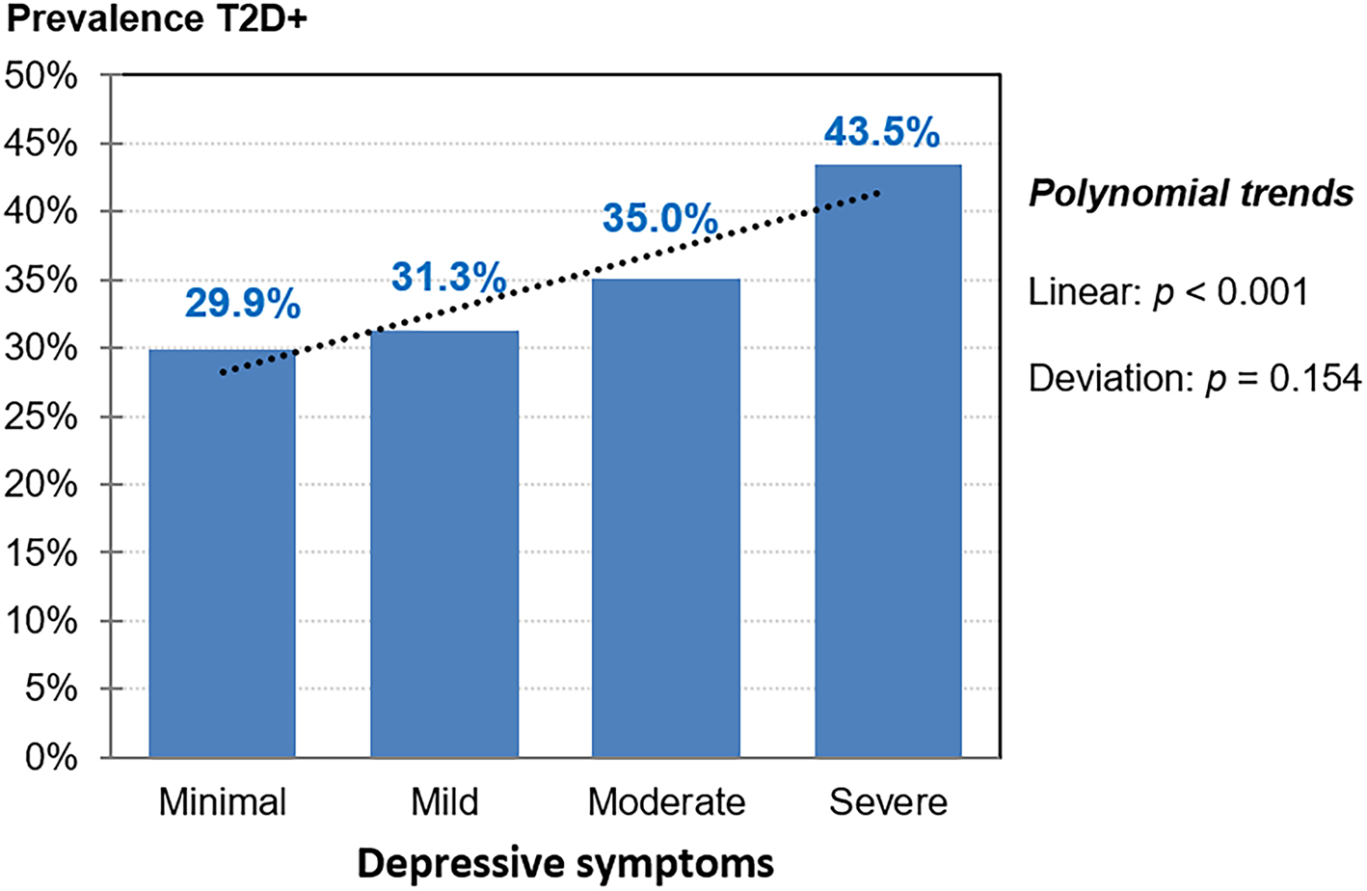
Bar chart with the prevalence of participants with T2D within each depressive symptom severity levels (cross-sectional analysis at baseline). Note: dash-line: linear trend. Sample size: *n* = 6224. T2D + : baseline prevalence of type 2 diabetes. Depressive symptoms, BDI-II scores: minimal (0–13), mild (14–19), moderate (20–28), and severe (29 or high)

An association was found between the presence/absence of T2D and the levels of depressive symptoms at baseline (*χ*^2^ = 15.84, *p* = 0.001). The polynomial contrast test showed a positive linear relationship (*χ*^2^ = 13.49, *p* = 0.001): participants with T2D reported more depressive symptoms compared to those without T2D (Table 2), and the higher the levels of depressive symptoms, the higher the prevalence of T2D (Fig. 1).

**Table 2:** Cross-sectional association between depressive symptom severity levels and T2D at baseline

Depressive symptoms	Type 2 diabetes				Chi-square tests	*χ*^2^(*df*)	*p*
	Absent (*n* = 4311)		Present (*n* = 1913)				
	*n*	%	*n*	%			
Severe	78	1.8	60	3.1	Pearson-test	15.84 (3)	0.001*
Moderate	280	6.5	151	7.9	Linear-by-linear	13.49 (1)	< 0.001*
Mild	473	11.0	215	11.2	Deviation	2.35 (2)	0.154
Minimal	3480	80.7	1487	77.7			

*df* degrees of freedom

*Bold: significant comparison. Depressive symptoms, BDI-II scores: minimal (0–13), mild (14–19), moderate (20–28), and severe (29 or high)

Table 3 shows the association between the levels of depressive symptoms at baseline and the sociodemographic and clinical measures of the study (analysis within the sub-sample of patients with T2D at baseline, *n* = 1913). Regarding the sociodemographic variables, higher levels of depressive symptoms were associated with sex (women), not being married, lower educational levels, and not being employed. Additionally, higher depressive symptoms were related to higher levels of HbA1c, cholesterol, glucose, and weight; also, to the use of treatment for T2D (metformin and insulin).

**Table 3:** Cross-sectional associations with the depressive symptom severity levels at baseline, within the sub-sample of participants with T2D at baseline

		Depressive symptoms (baseline)														*p*
		Minimal (*n* = 1487)			Mild (*n* = 215)				Moderate (*n* = 151)			Severe (*n* = 60)				
		*n*	%		*n*		%		*n*		%	*n*		%		
Sex																
Men		933	62.7		83		38.6		49		32.5	14		23.3		0.001*
Women		554	37.3		132		61.4		102		67.5	46		76.7		
Marital status																
Single		77	5.2		11		5.1		6		4.0	7		11.7		0.003*
Married		1177	79.2		149		69.3		113		74.8	40		66.7		
Divorced-separated		108	7.3		22		10.2		10		6.6	5		8.3		
Widowed		125	8.4		33		15.3		22		14.6	8		13.3		
Education																
Higher education		326	21.9		35		16.4		24		15.9	3		5.0		0.001*
Secondary		449	30.2		58		27.0		30		19.9	9		15.0		
Primary		705	47.4		120		55.8		96		63.6	48		80.0		
Less than primary		7	0.5		2		0.9		1		0.7	0		0.0		
Employment																
Employed		292	19.6		34		15.8		31		20.5	7		11.7		0.001*
Work at home		170	11.4		42		19.5		35		23.2	18		30.0		
Retired		938	63.1		123		57.2		75		49.7	31		51.7		
Unemployed		87	5.9		16		7.4		10		6.6	4		6.7		
Weight status																
Overweight (BMI 25–30)		390	26.2		49		22.8		33		21.9	6		10.0		0.001*
Obese I (BMI 30–35)		734	49.4		105		48.8		62		41.1	31		51.7		
Obesity II or more (BMI > 35)		363	24.4		61		28.4		56		37.1	23		38.3		
HbA1c																
Low-normal (< 7.0%)		925	62.2		127		59.1		83		55.0	28		46.7		0.034*
High (≥ 7.0%)		562	37.8		88		40.9		68		45.0	32		53.3		
Insulin for T2D																
No		1289	86.7		174		80.9		121		80.1	39		65.0		0.001*
Yes		198	13.3		41		19.1		30		19.9	21		35.0		
Duration of T2D																
Less than 1 year		133	8.9		17		7.9		11		7.3	5	8.3			0.980
Between 1 and 5 years		411	27.6		58		27.0		40		26.5	15	25.0			
More than 5 years	943			63.4		140		65.1		100	66.2	40	66.7			

	Mean	SD	Mean	SD	Mean	SD	Mean	SD	*p*
Total cholesterol, mg/dl	180.4	35.9	187.8	39.3	181.9	40.3	185.9	45.1	0.040*
LDLc, mg/dl	106.3	33.9	112.8	34.8	107.2	36.2	113.1	44.9	0.046*
HDLc, mg/dl	45.2	11.1	47.3	11.9	46.4	10.5	48.4	11.8	0.010*
Triglycerides, mg/dl	158.5	76.5	156.4	71.5	155.5	63.1	156.6	83.1	0.951
Fasting plasma glucose, mg/dl	138.0	34.1	136.6	33.7	139.8	37.4	150.6	37.3	0.035*
HbA1c, %	6.83	0.93	6.86	0.92	7.00	0.95	7.26	1.01	0.002*
Insulin mcU/ml	18.3	8.6	18.3	8.3	18.8	8.8	18.6	9.2	0.933
Systolic blood pressure, mm Hg	142.2	17.8	140.0	17.1	140.1	16.9	144.6	20.2	0.113
Diastolic blood pressure, mm Hg	81.0	10.3	80.1	9.9	80.1	10.6	78.9	10.9	0.246
Waist circumference, cm	109.6	9.3	108.3	9.4	109.6	8.9	109.9	11.1	0.256

*P* values are based on the difference between severity levels of baseline depressive symptoms (ANOVA for the continuous variables and *χ*^2^ test for categorical variables). HbA1c, glycosylated hemoglobin; HDLc, high‐density lipoprotein cholesterol; LDLc, Low‐density lipoprotein cholesterol. Depressive symptoms, BDI-II scores: minimal (0–13), mild (14–19), moderate (20–28), and severe (29 or high). HbA1c: low-normal (< 7%), high (≥ 7%)

*Bold: significant comparison

### Longitudinal analyses

Table 4 shows the frequency distribution of the depressive symptom levels at baseline and HbA1c at 1-year follow-up (analysis within the sample with T2D at baseline, *n* = 1694). Logistic regression adjusted for baseline HbA1c, the recruitment center and the intervention group showed a predictive association: the higher the depressive symptoms levels at baseline, the higher the likelihood of being within the high-range level of HbA1c. The association was obtained for an univariate model (with no adjustment: Wald-*χ*^2^ = 18.77, df = 3, *p* < 0.001), and for some alternative models with different covariates: (a) model 2 adjusted for HbA1c at baseline, center, and intervention group (Wald- *χ*^2^ = 22.27, df = 3, *p* < 0.001); (b) model 3 adjusted for HbA1c at baseline, center, intervention group, sex, age, education and incomes (Wald- *χ*^2^ = 24.62, df = 3, *p* < 0.001); and (c) model 4 adjusted for HbA1c at baseline, center, intervention group, and use of T2D medication (Wald- *χ*^2^ = 24.06, df = 3, *p* < 0.001).

**Table 4:** Longitudinal association between the depressive symptom severity levels at baseline and the HbA1c at 1-year follow-up, within the sub-sample of participants with T2D at baseline: logistic regression/ANCOVA

HbA1c (1-year follow-up)	Depressive symptoms (baseline)											
	Minimal (*n* = 1337)		Mild (*n* = 188)		Moderate (*n* = 121)		Severe (*n* = 48)		Model 1	Model 2	Model 3	Model 4
HbA1c %	Mean	SD	Mean	SD	Mean	SD	Mean	SD	*p*	*p*	*p*	*p*
	6.72	1.00	6.93	1.23	6.88	0.93	7.07	1.23	0.004*	0.011*	0.009*	0.008*
	*n*	%	*n*	%	*n*	%	*n*	%	*p*	*p*	*p*	*p*
Low-normal (< 7.0%)	917	68.6	105	55.9	69	57.0	27	56.3	0.001*	0.001*	0.001*	0.001*
High (≥ 7.0%)	420	31.4	83	44.1	52	43.0	21	43.8				

Model 1: crude results (no adjustment). Model 2: adjusted for HbA1c at baseline, center, and intervention group. Model 3: adjusted for HbA1c at baseline, center, intervention group, sex, age, education, and incomes (0: unemployed without incomes versus 1: employed or unemployed with incomes). Model 4: adjusted for HbA1c at baseline, center, intervention group, and use of T2D medication (metformin and/or insulin). HbA1c: glycosylated hemoglobin. Depressive symptoms, BDI-II scores: minimal (0–13), mild (14–19), moderate (20–28), and severe (29 or high). HbA1c: low-normal (< 7%), high (≥ 7%)

*Bold: significant comparison

### Path analysis

**Fig. 2:**
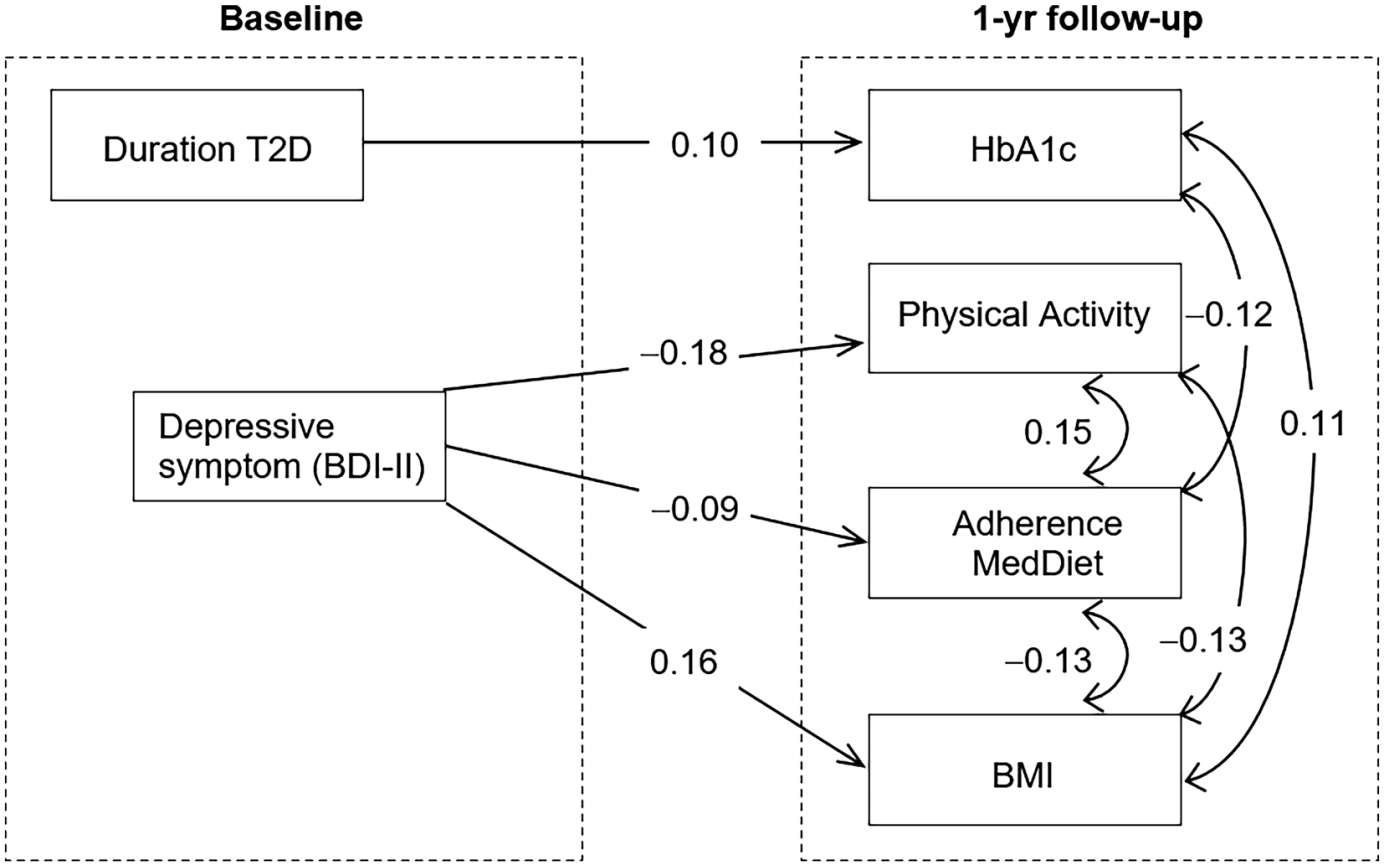
Standardized coefficients in the path analysis, within the sub-sample of patients with T2D at baseline. Note. HbA1c: glycosylated hemoglobin. Physical activity: energy expenditure in leisure-time physical activity. Adherence MedDiet: adherence to MedDiet intervention. BMI: body mass index (Kg/m^2^). Depressive symptoms: BDI-II total score. Model adjusted for sex, age, use of T2D medication HbA1c at baseline, center, and intervention group. Only significant results retained in the model. Sample size: *n* = 1694

Figure 2 shows the path diagram with the standardized coefficients obtained in the SEM (analysis within the T2D + group at baseline, *N* = 1,694; complete results are showed in Table S1, supplementary) Adequate goodness of fit was obtained (RMSEA = 0.02, CFI = 0.998, TLI = 0.999, and SRMR = 0.060), and the global predictive capacity was approximately 58% (CD = 0.415). After adjustment for sex, age, HbA1c at baseline, the center, and the intervention group and the use of T2D medication (metformin and/or insulin), it was found that higher HbA1c was predicted by longer duration of the T2D. Higher levels of depressive symptoms at baseline also predicted lower level of energy expenditure in leisure-time physical activity, lower likelihood of adherence to MedDiet, and higher BMI. At 1-year of follow-up, HbA1c was positively correlated with the BMI and negatively correlated with the likelihood of adherence to MedDiet, the BMI was also negatively correlated with the energy expenditure in leisure-time physical activity and the likelihood of adherence to MedDiet. Energy expenditure in leisure-time physical activity was positively correlated with adherence to MedDiet. Table S1 shows complete results for the SEM.

## Discussion

Aligned with our initial hypotheses, depressive symptoms appeared more frequently in older adults with MetS who presented T2D. While minimal-to-mild depressive symptoms were predominantly reported in the total sample, there was a significantly higher proportion of individuals referring moderate and severe depressive symptoms in the T2D group. Among them, we found that higher levels of depressive symptoms were associated with worse baseline metabolic state. A higher prevalence of depressive symptoms has been previously described in individuals with T2D compared to the general population [8, 31]. According to our results, this finding could be replicated in individuals with MetS, suggesting that T2D may represent an independent risk factor among them for the development and severity of these psychological traits, even without meeting criteria for a clinical depression [32]. While, in most cases, a positive screening would not necessarily imply the presence of a psychiatric disorder [4, 31], subthreshold depressive symptoms have an impact on health outcomes, premature mortality, and burden in T2D [8, 32]. In this context, our findings highlight the need to monitor and carefully manage depressive symptoms in older age, when factors such as age and other chronic somatic diseases confer an increased metabolic and emotional fragility [7]. Different neurobiological and environmental factors may contribute to explain the association between T2D and depressive symptoms, as well as the association between these psychological traits and a poorer metabolic control.

From a biophysiological perspective, common mechanisms may underlie the complex association between T2D and mood disturbances, mutually influencing their development, clinical course, and management [33]. Mainly, a dysfunctional central insulin signaling due to peripheral insulin resistance, immune-inflammatory pathways, and the hyperactivation of the HPA axis are considered key interconnected neurobiological mechanisms [33–35]. Resulting alterations in neurogenesis and neuroplasticity [36] have been associated with both depressive symptoms and glycemic control [33, 37]. Increased levels of glucocorticoids, catecholamines, and inflammatory biomarkers in individuals with T2D and depressive symptoms could exacerbate not only insulin resistance, but also lipolysis with an increase in circulating free fatty acids, BMI, and atherosclerotic processes [38]. Hence, these neurobiological disturbances might also play a role in the relationship between depressive symptoms and worse global metabolic state in individuals with MetS and T2D. On the other hand, obesity and other metabolic disturbances, such as dyslipidemia, could also contribute to mood and glucose disturbances [13]. Interestingly, related-genetic disturbances, such as those affecting HPA axis, neurotransmitter signaling, endocrine factors, such as leptin, or circadian rhythms, have been described in T2D and mood disturbances [39]. Indeed, a moderate co-heritability has been described between depression and cardiovascular disturbances, including blood pressure and serum lipid levels [39]. The interaction of genetics with environmental factors related to stress, physical activity, diet, and other lifestyle features could influence the progression and pathogenesis of both cardiometabolic and mood disturbances through epigenetic mechanisms [39].

When we focused on the sub-sample with T2D, our results showed a significant impairment in metabolic control from mild depressive symptom levels at both baseline and at 1-year of follow-up. To date, previous research has shown that while a clearer relationship between depressive symptoms and poorer glycemic control has been established in underage population [40], mixed results have been reported from different cross-sectional and longitudinal studies in adult samples [4]. Some research supports that diabetes-related distress would be the psychological trait particularly associated with glycemic control, and has been proposed as a better predictor of metabolic control than depressive-related disturbances [4, 41]. Alternatively, consistent with our findings, some studies have suggested that depressive symptoms may be independently related to self-care and diabetes self-management [32, 35]. In line with these findings, individuals with T2D who experienced depressive symptoms have reported missing medical appointments, lower adherence to diet and exercise guidelines, a disrupted use of medication, as well as a lower monitoring of glycemia and physical complications [34, 42]. In addition to the association between depressive symptoms and a poorer metabolic control, our results support the need to consider a quantitative measure of symptom severity. Accordingly, a study in individuals with T2D found that merely a 1-point increase in depressive symptom scores evaluated trough the Harvard Department of Psychiatry/National Depression Screening Day Scale (HANDS) resulted in a 10% increased risk of nonadherence to fruit and vegetable intake and foot care [42]. In this vein, it has been hypothesized that the presence and increase of depressive symptoms could represent a marker of increased health risk among patients with T2D [31]. Hence, the optimization of preventive and therapeutic approaches for depressive symptoms in T2D since its early stages seems crucial, considering the dynamic impact of depressive symptoms on the metabolic control in the short and middle term. In this regard, the early identification of patients at risk for T2D could be helpful. In our findings, the sociodemographic profile of individuals with higher levels of depressive symptoms agrees with the previous literature defining shared risk factors for T2D and mood disturbances, such as female sex, single marital status, and low socioeconomic income [35]. Individuals with more disadvantageous socioeconomic circumstances might experience less external care and more difficult accessibility to treatment. This scenario could influence a poorer self-care and diabetes management, as well as the presence of higher emotional disturbances which, in turn, may contribute to a poorer metabolic control [31].

Remarkably, we found that higher levels of depressive symptoms at baseline were predictive of lower adherence to MedDiet and higher BMI. Previous studies have shown that individuals with depressive symptoms have more unhealthy eating patterns, and that higher levels of symptoms are associated with emotional eating, as with uncontrolled eating, which promotes weight gain [43]. It is very common for people to turn to emotional eating as strategy to cope with stress or anxiety, sometimes along with increased appetite due to the use of some psychotropic medications, people resort to emotional eating as a strategy to cope with these issues [44]. In turn, people with diabetes generally feel that nutritional issues are the most complex in managing their disease [45]. Research has shown that when assessing diabetes-related emotional distress, some of the most common concerns are shame and guilt related to lifestyle, including food choices, and stigma related to obesity [46]. Another barrier expressed by people with diabetes is the difficulty in maintaining the motivation for self-care that diabetes and obesity require, and the need for support to do so [45]. Low self-efficacy in people with comorbid mental health problems and diabetes has been associated with lower engagement in diabetes self-management activities [47]. This highlights the importance of improving self-efficacy as a key component of diabetes self-management. Motivational interviewing techniques, patient-centered approaches based on dynamic personal goals, and action planning have been found to be useful strategies for maintaining and improving motivation [48], and its implementation has shown improvements in dietary management and self-monitoring of blood glucose [49]. As a goal of nutritional therapy for adults with diabetes, the ADA recommends promoting healthy eating patterns that enhance the consumption of nutrient-dense foods, such as the MedDiet, through practical tools to achieve glycemic control and delay diabetes complications [26]. Similarly, these guidelines emphasize not only addressing patients’ personal and cultural preferences, but also their willingness and ability to make behavioral changes and their existing barriers to change.

Likewise, our results also showed that higher levels of depressive symptoms at baseline were a predictor of the lower levels of energy expenditure in leisure-time physical activity which, in turn, influenced BMI. Mental disturbances have been associated with reduced physical activity [50]. Some psychological symptoms such as loss of interest and lack of motivation, as well as the sedating effects of some psychiatric medications, can significantly reduce both planned and daily physical activity. Besides, disrupted sleep patterns, altered circadian rhythms, or anhedonia have been related to sedentary lifestyle favoring insulin resistance [51], an increased BMI, and higher cardiovascular risk, potentially interfering with metabolic control [52]. Moreover, many people with mood disturbances report feeling uncomfortable in noisy and crowded environments, such as gyms, or in open or enclosed spaces [53]. In this line, anxiety traits and social isolation may also interfere with a physically active lifestyle due to reduced social incentive motives for diabetes care management [52]. Thus, physical activity is a fundamental pillar in the treatment of both T2D and mental health disturbances. Recognizing the limitations of people with mental health problems can help to better intervene throughout the treatment. Regular exercise has several well-reported physical benefits for people with T2D, including improvements in cardiovascular fitness, reductions in blood pressure and body weight, and improvements in diabetes management [26]. Furthermore, exercise can produce many psychological changes, positively affecting the mood state and self-esteem, and reducing anxiety and stress levels [54].

### Final thoughts: considerations for primary care

A more holistic and integrative approach may be needed in an intent to encompass both metabolic management and depressive symptoms in individuals with T2D, as the improvement of one will influence the other. In this context, a trained multidisciplinary team seems to be crucial [31]. Although the management of depressive symptoms in these patients requires the involvement of physicians from different spcialities, other health care providers, such as nurses and psychologists, should also be involved [35]. The ADA, in their 2023 Standards of Care in Diabetes for Primary Care Providers, recommends that psychosocial care should be integrated into routine medical care for all people with diabetes [26]. Routine assessment and evaluation of mood-related disturbances, such as depressive symptoms and diabetes-related distress, should be included in every visit as part of comprehensive care. That said, well-validated scales should be used to avoid under- and overestimation mood-related symptoms as specific therapeutic approaches may be required [35]. When warranted, patients should be referred to mental health facilities for more formal diagnostic assessments and specific interventions as needed. In these cases, it is critical that mental health professionals treating people with diabetes address specific therapeutic goals related to diabetes management, as there is growing evidence that psychological interventions are most effective when tailored specifically to people with diabetes [55]. Due to the complexity of the pathology and to provide effective care, international diabetes-related institutions recommend that mental health professionals have expertise in the unique issues associated with this condition [56]. Scientific evidence has shown that mental health interventions can successfully target and modify aspects of diabetes care to improve outcomes associated with standard care. Evidence suggests that identifying alternative, realistic goals, such as improved adherence and glycemic control, fewer hospital admissions, fewer episodes of diabetic ketoacidosis, and improved attendance at medical appointments, can have a significant impact on patients’ physical and mental health, reducing the development of complications and improving their quality of life [26]. Given the role that lifestyle factors play in the onset and course of both T2D and mood disturbances, they should be considered crucial therapeutic targets [57].

### Limitations

This study must be interpreted considering some limitations. First, the characteristics of the study cohort (i.e., older adults with MetS and high cardiovascular risk) limit the extrapolation of the results to the general population. Second, our analyses were not based on a clinical diagnosis of depression but on a screening tool for depressive symptoms (BDI-II). However, this is a well-validated questionnaire and widely used instrument. Third, the self-reported nature of the data collection may affect the reliability of the study. Fourth, although our study is based on a large sample, the longitudinal analysis is limited by some dropouts and incomplete data during follow-up, which could represent a potential selection bias. Similarly, the consolidation of our results may require longer follow-up periods. Finally, as in any observational study, the possibility of unmeasured confounding could not be excluded. Nonetheless, relevant confounders were considered throughout the analyses.

## Conclusions

The present study suggests an association between the presence of T2D and the severity of depressive symptoms, which may be of particular importance in vulnerable populations such as older adults with MetS. Our results highlight the importance of monitoring mood disturbances, as their presence has been shown to interfere with metabolic control even from mild levels of severity, both in the short and middle term. In this line, our study also supports the idea of depressive symptom severity as a predictive factor of poorer metabolic control, throughout its association with lower diabetes care and higher BMI. Therefore, primary care physicians and other specialists directly involved in the management of T2D need to be familiar with useful and well-validated screening tools for mood disturbances, that should be incorporated as part of the routine examination of these patients. Ensuring a well-established coordination network between different health care providers, including mental health care professionals, allows personalized psychosocial needs to be integrated into physical health care, either from a comprehensive or parallel perspective. In T2D, including psychoeducation, motivational, and goal-oriented psychological interventions could be especially helpful in the management of metabolic and depressive features.

### Supplementary Information

Below is the link to the electronic supplementary material.

Supplementary file1 (DOCX 103 KB)

## Data Availability

Due to signed consent agreements regarding data sharing, there are restrictions on data availability for the PREDIMED-Plus trial. These only allow access to external researchers for studies following the project purposes. Requestors wishing to access the PREDIMED-Plus trial data used in this study can make a request to the PREDIMED-Plus trial Steering Committee chair: predimed_plus_scommittee@googlegroups.com.
