# Supplementary material for: Association between type 2 diabetes and depressive symptoms after a 1-year follow-up in an older adult Mediterranean population
Source: J Endocrinol Invest. 2024 Jan 13;47(6):1405–18. doi: 10.1007/s40618-023-02278-y (PMC11142971; doi:10.1007/s40618-023-02278-y)

**Title:** Association between type 2 diabetes and depressive symptoms after a 1-year follow-up in an older adult Mediterranean population.

**Journal:** Journal of Endocrinological Investigation

**Authors:** Isabel Baenas^1,2,3,4#^; Lucía Camacho-Barcia^1,2,3#^; Roser Granero^2,3,5^; Cristina Razquin^2,6^; Dolores Corella^2,7^; Carlos Gómez-Martínez^2,8,9^; Olga Castañer-Niño^10,11^; J Alfredo Martínez^2, 12,13^; Ángel M. Alonso-Gómez^2,14^; Julia Wärnberg^2,15^; Jesús Vioque^11,16^; Dora Romaguera^2,17^; José López-Miranda^2,18^; Ramon Estruch^2,19^; Francisco J Tinahones^2,20^; José Lapetra^2,21^, J. Luís Serra-Majem^2,22^; Naomi Cano-Ibáñez^11,23,24^; Josep A. Tur^2,25^; Vicente Martín-Sánchez^11,26^; Xavier Pintó^2,27^; José Juan Gaforio^11,28^; Pilar Matía-Martín^29^; Josep Vidal^30,31^; Clotilde Vázquez^2,32^, Lidia Daimiel^2,33^, Emilio Ros^2,34^; Susana Jiménez-Murcia^1,2,3,35^; Søren Dalsgaard^36,37,38^; Ana Garcia-Arellano^2,6^; Nancy Babio^2,8,9^**; Jose V. Sorli^2,7^;** Camile Lassale^2,39^; **Antonio Garcia-Rios^2^**^,18^**;** Manuela García-de-la-Hera^11,16^**; Enrique Gómez-García^2,15^; M Ángeles Zulet^2,12,13^;** Jadwiga Konieczna^2,17^**;** Sandra Martín-Peláez^11,23^**;** Lucas Tojal-Sierra^2,14^**;**Francisco Javier Basterra-Gortari^2,6,40^; Sara de las Heras-Delgado^2,8,9^**; Olga Portoles^2,6^;** Miguel Ángel Muñoz-Pérez^41^**;** Antonio P. Arenas-Larriva^2,18^**;** Laura Compañ-Gabucio^11,16^**;**Sonia Eguaras^6^; Sangeetha Shyam^2,8,9^**;** Montserrat Fitó^2,10^; Rosa M. Baños^2,42^; Jordi Salas-Salvadó**^2,8,9*^** & Fernando Fernández-Aranda^1,2,3,35^*

**Corresponding authors:**

Jordi Salas‐Salvadó, Human Nutrition Unit, Faculty of Medicine and Health Sciences, Universitat Rovira I Virgili, C/ Sant Llorenç 21, 43201 Reus, Spain. ORCID: 0000-0003-2700-7459. Email: [jordi.salas@urv.ca](mailto:jordi.salas@urv.ca)t

-Centro de Investigación Biomédica en Red, Fisiopatología de la Obesidad y la Nutrición (CIBERobn), Instituto de Salud Carlos III, 28029 Madrid, Spain.
-Universitat Rovira i Virgili, Departament de Bioquímica i Biotecnologia, Grup ANUT-DSM Unitat de Nutrició Humana, 43201 Reus, Spain.
-Institut d'Investigació Sanitària Pere Virgili (IISPV), 43007 Reus, Spain.

Fernando Fernández-Aranda. Eating Disorders Unit, Clinical Psychology Unit, University Hospital of Bellvitge-IDIBELL and CIBEROBN, Feixa Llarga s/n 08907 Hospitalet de Llobregat (Barcelona, Spain). Tel. +34-93-2607227, Fax. +34-93-2607193. ORCID: 0000-0002-2968-9898. Email: [ffernandez@bellvitgehospital.cat](mailto:ffernandez@bellvitgehospital.cat)

-Clinical Psychology Unit, University Hospital of Bellvitge, 08907 Barcelona, Spain.
-Centro de Investigación Biomédica en Red, Fisiopatología de la Obesidad y la Nutrición (CIBERobn), Instituto de Salud Carlos III, 28029 Madrid, Spain.

-Psychoneurobiology of Eating and Addictive Behaviors Group, Neurosciences Programme, Bellvitge Biomedical Research Institute - IDIBELL, 08908, Barcelona, Spain.

-Department de Psicobiologia i Metodologia de les Ciències de la Salut, Universitat Autònoma de Barcelona, 08193 Barcelona, Spain.

**Supplementary Material**

***Table S1.*** Complete results for the SEM

| Structural |  | *Coeff.* | *SE* | *z-stat* | *p* | *95%CI coefficient* | |
| --- | --- | --- | --- | --- | --- | --- | --- |
| HbA1c – 1 year | Duration T2D - baseline | 0.1022 | 0.0193 | 5.29 | <.001 | 0.0643 | 0.1401 |
| Physical activity – 1 year | Depressive symptoms- baseline | -0.1829 | 0.0235 | -7.79 | <.001 | -0.2289 | -0.1368 |
| Adherence MedDiet – 1 year | Depressive symptoms - baseline | -0.0871 | 0.0239 | -3.64 | <.001 | -0.1340 | -0.0401 |
| BMI – 1 year | Depressive symptoms - baseline | 0.1593 | 0.0236 | 6.76 | <.001 | 0.1131 | 0.2056 |
| Covariances – 1year | HbA1c – Adherence MedDiet | -0.1159 | 0.0256 | -4.52 | <.001 | -0.1661 | -0.0656 |
|  | HbA1c - BMI | 0.1072 | 0.0240 | 4.46 | <.001 | 0.0601 | 0.1543 |
|  | Physical activity - Adherence MedDiet | 0.1530 | 0.0236 | 6.48 | <.001 | 0.1068 | 0.1992 |
|  | Physical activity - BMI | -0.1340 | 0.0237 | -5.64 | <.001 | -0.1805 | -0.0875 |
|  | Adherence MedDiet - BMI | -0.1285 | 0.0239 | -5.38 | <.001 | -0.1753 | -0.0817 |

*Note.* BMI, body mass index; HbA1c, glycated hemoglobin; MedDiet, Mediterranean Diet; T2D, type 2 diabetes. Coeff: standardized coefficient.

**Figure S1.** Flow-chart with the sampling procedure

*Note.* T2D: type 2 diabetes


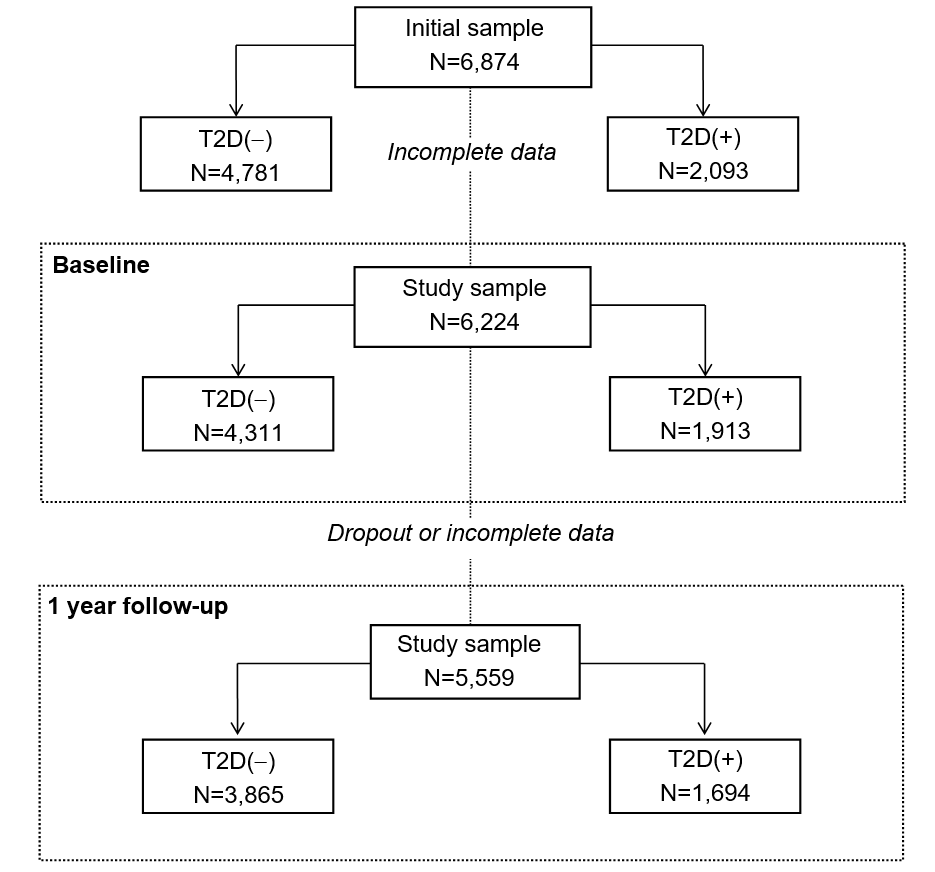

Supplement: Supplementary file 1 — Supplementary file1 (DOCX 103 KB) [file 40618_2023_2278_MOESM1_ESM.docx]
